# EHMTI-0102. Prospective analysis of the use of onabotulinumtoxina (botox®) In the treatment of chronic migraine; real-life data in 299 patients from hull, UK

**DOI:** 10.1186/1129-2377-15-S1-G19

**Published:** 2014-09-18

**Authors:** M Khalil, H Zafar, V Quarshie, F Ahmed

**Affiliations:** 1 Neurology, Hull Royal Infirmary, Wakefield, UK

## Background

Chronic migraine (CM) affects 2% of the general population with substantial impact on quality of life. The efficacy and safety of Botox in CM was confirmed in the phase III Research Evaluating Migraine Prophylaxis Therapy (PREEMPT) clinical programme. Despite this, few data exist in the real-life setting.

## Aim

To evaluate the efficacy of Botox in adults with CM in real-life setting.

## Method

Adult patients with CM attending the Hull migraine clinic were offered Botox based on clinical needs using the PREEMPT protocol. Headache diaries were maintained for at least 30 days prior to and continuously after Botox (July 2010 and January 2014). Data were extracted for headache, migraine, and headache-free days. A responder was defined as one with either a 50% reduction in either headache days or migraine days an increment in crystal clear days twice that of the baseline.

## Results

Of a series of 331 patients, full data were available on 299 patients There was significant decrement in headache and migraine days as well as similarly significant increment in headache-free days. Responder rate was calculated for the above mentioned 3 categories.

## Discussion

Our analysis has shown that, in a real-life clinical setting, Botox can effectively reduce headache and migraine days, and increase crystal clear days from baseline. Our cohort represent a more severely affected population than seen in PREEMPT study; furthermore the pre-treatment headache days’ number was higher in our patients . We are hoping to present the data on 500 patients as we continue to treat patients.

